# DFT Calculations for Mössbauer Properties on Dinuclear Center Models of the Resting Oxidized Cytochrome *c* Oxidase

**DOI:** 10.1002/cphc.202100831

**Published:** 2022-03-01

**Authors:** Wen-Ge Han Du, Andreas W. Götz, Louis Noodleman

**Affiliations:** [a] Department of Integrative Structural and Computational Biology, The Scripps Research Institute, 10550 North Torrey Pines Road, La Jolla, CA 92037, USA; [b] San Diego Supercomputer Center, University of California San Diego, 9500 Gilman Drive MC0505, La Jolla, CA 92093, USA

**Keywords:** cytochrome *c* oxidase, resting state, Mössbauer, isomer shift, quadrupole splitting

## Abstract

Mössbauer isomer shift and quadrupole splitting properties have been calculated using the OLYP-D3(BJ) density functional method on previously obtained (Han Du W–G, et al. *Inorg Chem*. 2020, *59*, 8906–8915) geometry optimized Fe_a3_^3+^−H_2_O−Cu_B_^2+^ dinuclear center (DNC) clusters of the resting oxidized (O state) “as-isolated” cytochrome *c* oxidase (C*c*O). The calculated results are highly consistent with the available experimental observations. The calculations have also shown that the structural heterogeneities of the O state DNCs implicated by the Mössbauer experiments are likely consequences of various factors, particularly the variable positions of the central H_2_O molecule between the Fe_a3_^3+^ and Cu_B_^2+^ sites in different DNCs, whether or not this central H_2_O molecule has H-bonding interaction with another H_2_O molecule, the different spin states having similar energies for the Fe_a3_^3+^ sites, and whether the Fe_a3_^3+^ and Cu_B_^2+^ sites are ferromagnetically or antiferromagnetically spin-coupled.

## Graphical Abstract

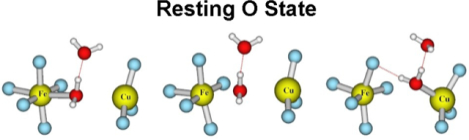

**Resting O State**: Mössbauer isomer shift and quadrupole splitting properties for the resting oxidized “as-isolated” **O**[Fe_a3_^3+^−H_2_O−Cu_B_^2+^] state of the dinuclear center (DNC) clusters of cytochrome *c* oxidase (C*c*O) have been calculated with the OLYP-D3(BJ) density functional method and have been compared with available experiments.

## Introduction

Cytochrome *c* oxidases (C*c*Os) are the terminal electron acceptors in the respiratory chain of mitochondria and many bacteria.^[1–3]^ These proteins reduce O_2_ to H_2_O and use the resulting energy to pump protons across the membrane. This produces the chemiosmotic proton gradient that is subsequently harnessed by ATP synthase to synthesize ATP.^[4–8]^ The catalytic site of C*c*O that binds and reduces O_2_ by 4e^−^/4H^+^ transfer contains a heme a_3_ (Fe_a3_) and a Cu (Cu_B_) ion that are in spatial vicinity (~5 Å distance). This Fe_a3_−Cu_B_ active site is usually called the dinuclear (or binuclear) center/complex (DNC or BNC). In all types of C*c*O enzymes, the iron in the Fe_a3_ site is coordinated to heme and an axial histidine ligand (His384, residue numbers in this paper are by default for *ba*_3_ C*c*O from *Thermus thermophilus* (*Tt*)). The copper in the Cu_B_ site is coordinated to three histidine ligands: His233, His282, and His283, where the His233 side chain is also covalently linked to the side chain of Tyr237. This special tyrosine side chain can be alternatively in neutral (Tyr−OH), deprotonated ionic (Tyr−O^−^), or radical (Tyr−O^•^) states, and plays an important role in electron and proton transfer in the DNC.^[9]^

**Figure 1.**
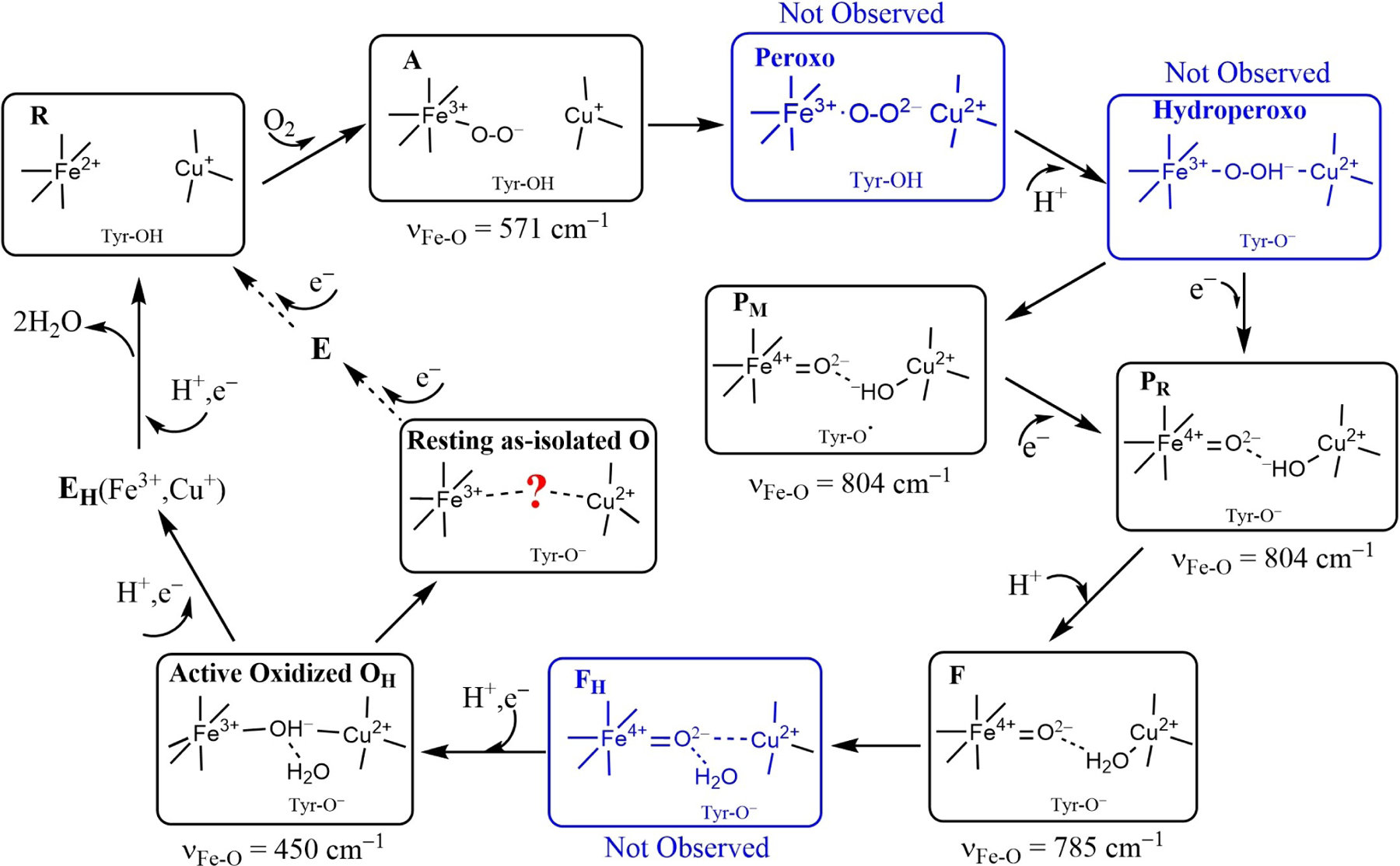
Feasible intermediate states of the DNC in the catalytic cycle, in which **A**, **P**_**M**_/**P**_**R**_, **F**, and **O**_**H**_ were identified by resonance Raman experiments,^[8,10,11]^ and their DNC’s are likely in the forms presented above.^[18]^ Although the states in the blue frames were not observed experimentally, they may exist for a short time in the catalytic cycle.

The oxidation, spin, and ligation states of the Fe_a3_ and Cu_B_ sites change during the catalytic cycle.^[8–19]^ Although many insights into the intermediate states of the DNC in the catalytic cycle have been obtained (Figure 1) (see review articles^[8,10,11]^ and the references therein), the detailed DNC structure of the resting oxidized as-isolated C*c*O state (state **O**) has been under debate for over 20 years despite various spectral and structural analyses that have been made.^[20–28]^

The electron density lying directly between Fe_a3_ and Cu_B_ in the as-isolated oxidized *aa*_3_ type C*c*Os from *Paracoccus denitrificans* (*Pd*) and *Rhodobacter sphaeroides* (*Rs*) was initially interpreted as a H_2_O and OH^−^ ligand pair.^[21,22]^ Later, stronger and more compact electron density for a peroxide type dioxygen species (O1–O2) bridging the Fe_a3_ and Cu_B_ DNC was observed in higher resolution X-ray crystal structures of the oxidized C*c*Os from *Pd* (PDB code: 3HB3, 2.25 Å resolution),^[23]^ from bovine heart (PDB code: 2ZXW, 1.95 Å resolution),^[24]^ and from *ba*_*3*_ *Tt* (3S8G and 3S8F, 1.8 Å resolution).^[25]^ Further, the peroxide type species in the resting oxidized DNC was also observed from analysis of an X-ray free-electron laser (XFEL) experiment (1.9 Å resolution),^[26]^ and very recently from analysis of a single-particle cryo-electron microscopy (cryo-EM) experiment at similar resolution.^[29]^ Different groups reported different O1–O2 distances from 1.4~1.7 Å. Recently, a single hydroxide or alternatively a single water molecule between the Fe_a3_ and Cu_B_ sites was reported in a radiation-damage-free oxidized *ba*_3_ C*c*O structure (2.3 Å resolution) at room temperature.^[27]^ However, analysis of very recent low-dose high-energy X-ray data on the oxidized-resting bovine heart C*c*O again showed a peroxide-shaped electron density between the Fe_a3_ and Cu_B_ sites.^[28]^ Even at fairly high resolution currently available (1.9 Å), there is considerable uncertainty in the electron density between the metals, and in the correct modeling of the corresponding ligands, which are not clearly known prior to the fits. There is, in practice, both static and dynamic disorder in the ligand positions, typically represented by isotropic B factors. There is further disorder in the Fe and Cu positions as well. (While the variation in the Fe and Cu positions is expected to be geometrically smaller, they have much higher electron densities than di-oxygen or water species.) These B factors are variables with a potential range of values during the density fitting, and influence the final electron densities found.

In order to examine what species lies between the Fe_a3_^3+^ and Cu_B_^2+^ sites producing the apparent extended di-oxygen type electron density in the DNC of the oxidized as-isolated C*c*O, previously we have performed DFT calculations^[30–32]^ on a series of DNC model clusters based on the X-ray crystal structure 3S8G^[25]^ from *Tt ba3* C*c*O. Our calculations have shown that the observed di-oxygen species cannot be represented by O_2_^2−^, O_2_^•−^, or H_2_O_2_, since the DFT optimized structures with bridging O_2_^2−^ or O_2_^•−^ have large structural discrepancies compared with the X-ray crystal structure, and the H_2_O_2_ is not stable between the Fe_a3_^2+^ and Cu_B_^+^ sites (Fe_a3_^3+^ and Cu_B_^2+^ metal sites were assumed reduced in the synchrotron X-ray beam).^[30]^ We initially came to the conclusion that the observed di-oxygen species was best represented as HO_2_^−^, which could be a product of the photoreaction of the H_2_O/OH^−^ ligands in Fe_a3_^3+^−H_2_O⋯OH^−^−Cu_B_^2+^/Fe_a3_^3+^−OH^−^⋯H_2_O−Cu_B_^2+^ type DNC structures with associated 2e^−^ transfer to the adjacent oxidized Fe_a3_^3+^ and Cu_B_^2+^ sites in the X-ray beam.^[30]^ However, if the resting oxidized DNC structure is originally (before X-ray irradiation) in the Fe_a3_^3+^−H_2_O⋯OH^−^−Cu_B_^2+^ or Fe_a3_^3+^−OH^−^⋯H_2_O−Cu_B_^2+^ form, the X-ray crystallographic experiments should still show H_2_O⋯OH^−^ as the dominant bridging species in the DNC with long O⋯O distance, 2.5 Å or greater. Because the X-ray crystal structure represents both a spatial and time average over many billions of enzyme molecules, while some effects due to the X-ray irradiation are probably observable with careful attention to the time course, these effects are not likely to be dominant averaged over billions of structural sites. These arguments are even stronger when applied to XFEL experiments, since the total radiation dose is much smaller than in synchrotron X-ray experiments, and the relevant time scale for diffraction is far shorter. Further, our very recent calculations have demonstrated that the Fe_a3_^3+^−H_2_O⋯OH^−^−Cu_B_^2+^/Fe_a3_^3+^−OH^−^⋯H_2_O−Cu_B_^2+^ type structures are also unlikely to represent the resting state of the DNC, consistent with the structural evidence and analysis above.^[32]^

**Figure 2.**
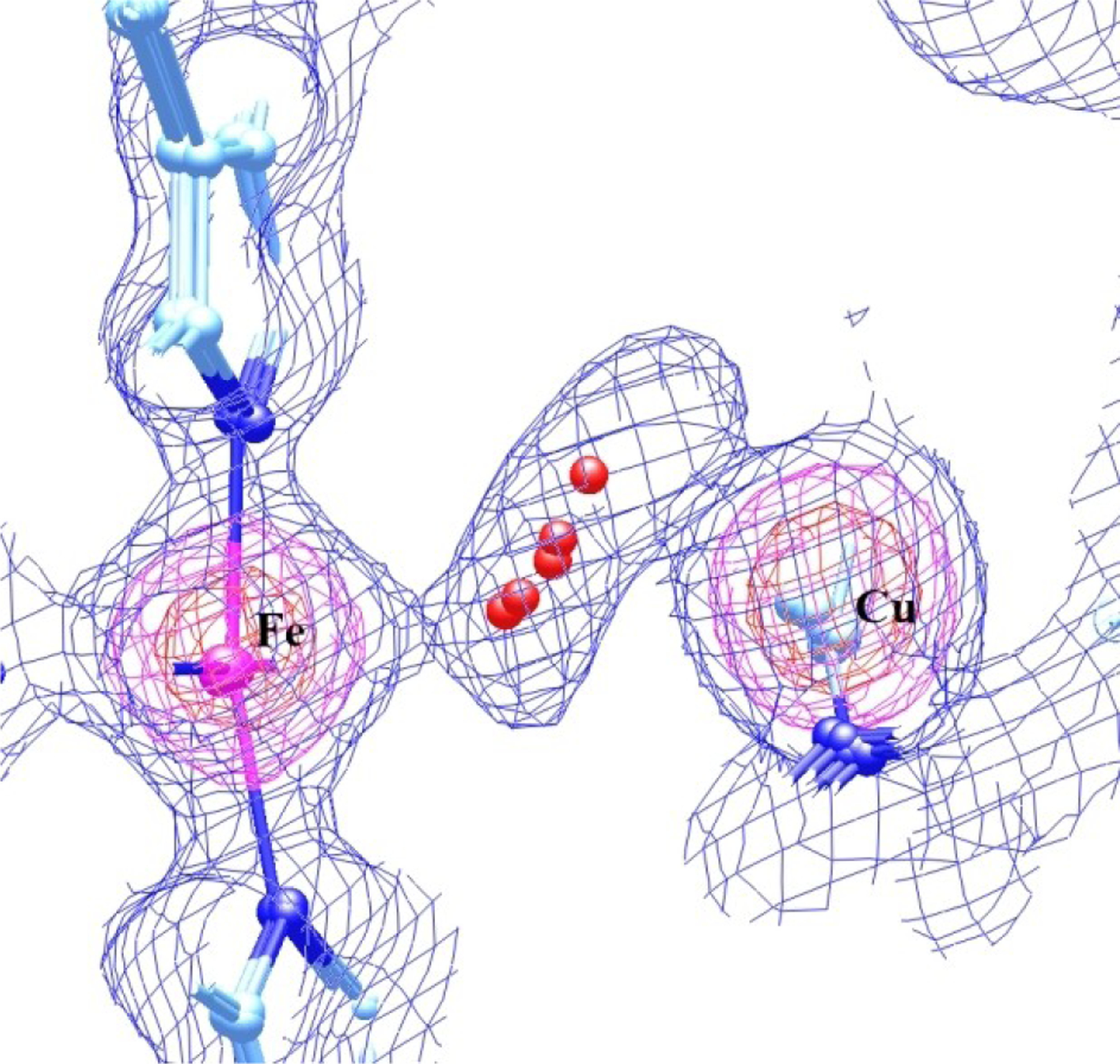
The overlap of the electron density map that was reconstructed from the oxidized as-isolated *Tt ba*_3_ X-ray crystal structure 3S8G^[25]^ data file with several of our calculated resting O state DNC structures, in which a water molecule (in red color) resides in different locations between the Fe_a3_^3+^ and Cu_B_^2+^ sites with very similar energies. Reprinted with permission from Figure 7 of Ref. [32], https://pubs.acs.org/doi/10.1021/acs.inorgchem.0c00724, copyright (2020) American Chemical Society (further permissions for reusing this figure should be directed to the ACS).

Our most recent calculations have shown that the observed peroxide type electron density between the two metal centers is probably a mistaken analysis due to superposition of the electron density of a water molecule located at alternative positions between Fe_a3_^3+^ and Cu_B_^2+^ sites in DNC’s of different C*c*O molecules.^[32]^ Our calculations indicate that the H_2_O molecule in the resting state **O**[Fe_a3_^3+^−H_2_O−Cu_B_^2+^] DNC structures can bind with either the Fe_a3_^3+^ or the Cu_B_^2+^ site, or can reside at several positions between the Fe_a3_^3+^ and Cu_B_^2+^ sites that are all energetically similar, depending on the Fe_a3_^3+^−Cu_B_^2+^ distance and H-bonding interaction with an additional H_2_O molecule. (In our modeling, the latter water lies well off the direct line between the Fe and Cu ions.)^[32]^ Figure 2 shows the overlap of the electron density map reconstructed from the X-ray crystal structure 3S8G^[25]^ of *Tt ba*_3_ and several of our geometry optimized **O**[Fe_a3_^3+^−H_2_O−Cu_B_^2+^] DNC structures with very similar energies and with a H_2_O molecule (in red) at different positions between the Fe_a3_^3+^ and Cu_B_^2+^ sites.^[32]^ Because the diffraction pattern and the inferred electron density map represents the effective long-range order averaged over a large number molecules and unit cells in the X-ray structure, this averaging can result in an apparent observed superposition of water at different positions between the Fe_a3_^3+^ and Cu_B_^2+^ metal sites.

Earlier ^57^Fe Mössbauer experiments also demonstrated extensive structural and electronic heterogeneities in the DNC for the resting **O** state C*c*Os.^[33–36]^ The major Mössbauer experimental observations on different C*c*Os are summarized in Table 1. Initial experiments on both *Tt c*_1_*aa*_3_ and bovine *aa*_3_ show broad ^57^Fe_a3_ spectra with isomer shift (δ) and quadrupole splitting (Δ*E*_Q_) values averaged at (δ=0.41 mm s^−1^, Δ*E*_Q_=1.10 mm s^−1^) and (δ=0.48±0.06 mm s^−1^, Δ*E*_Q_=1.0±0.1 mm s^−1^), respectively.^[33,34]^ These particular values show that the Fe_a3_^3+^ site is in a high-spin (HS) state. Later, more complicated Mössbauer spectra were reported for *Tt c*_1_*aa*_3_ at different pH,^[35]^ which showed that at least three HS−Fe_a3_^3+^ species existed at pH=5.7, and at least two HS−Fe_a3_^3+^ complexes were observed at pH=6.5, 7.8, and 9.3.^[35]^ However, only one set of the HS parameters was defined at (δ=0.41 mm s^−1^, Δ*E*_Q_=1.3 mm s^−1^). In addition, a “low-spin” (LS) Fe_a3_^3+^ species was identified at pH=5.7, 7.8, and 9.3 with the parameters (δ=0.29 mm s^−1^, Δ*E*_Q_=2.21 mm s^−1^).^[35]^ Note that this Fe_a3_^3+^ species may be alternatively in an intermediate-spin (IS) state based on the observed isomer shift and quadrupole splitting values. Further, more complex Fe_a3_^3+^ species which were temperature-dependent in *Tt ba*_3_ were also observed in Mössbauer experiments.^[36]^ Briefly, a HS−Fe_a3_^3+^ species with (δ=0.41 mm s^−1^, Δ*E*_Q_=0.71 mm s^−1^) and a LS Fe_a3_^3+^ (which may be an IS state instead) with (δ=0.29 mm s^−1^, Δ*E*_Q_=2.24 mm s^−1^) coexisted at 4.2 K.^[36]^ When the temperature was increased above 190 K, the “LS−Fe_a3_^3+^ species” began to transform to a different HS species with Δ*E*_Q_ ≈ 1 mm s^−1^. The δ value of this HS- Fe_a3_^3+^ species was not specifically reported. But from the context, we assume that it had the same value of δ=0.41 mm s^−1^ as the other HS species. (Otherwise, the fit for the new HS species would have required a different isomer shift value from the other HS state.) The LS→HS transformation is finished at 245 K. Therefore, the two different HS−Fe_a3_^3+^ species coexisted at 245 K.^[36]^

In our recent publication,^[32]^ we have calculated **O** state Fe_a3_^3+^−H_2_O−Cu_B_^2+^ DNC structures with Fe_a3_^3+^ in HS, IS, and LS states. The Fe_a3_^HS,3+^−H_2_O−Cu_B_^2+^ results were presented in the main text, while the Fe_a3_^IS,3+^−H_2_O−Cu_B_^2+^ and Fe_a3_^LS,3+^−H_2_O−Cu_B_^2+^ results have been given in the Supporting Information.^[32]^ Further, our calculations have also shown that the spin coupling between the Fe_a3_^3+^ and Cu_B_^2+^ sites appears very weak. For a given **O** state Fe_a3_^3+^−H_2_O−Cu_B_^2+^ DNC structure, similar energies are obtained whether Fe_a3_^3+^ Cu_B_^2+^ are ferromagnetically (F) or anti-ferromagnetically (AF) coupled. In the current paper, based on the geometries we obtained for the **O**[Fe_a3_^HS/IS/LS,3+^−H_2_O−Cu_B_^2+^] DNC structures,[32] we will calculate their ^57^Fe_a3_^3+^ Mössbauer isomer shift and quadrupole splitting properties and see how the calculated values correlate with the experimental observations. In contrast to the X-ray structures derived using synchrotron X-ray beams, or XFEL pulses, Mossbauer gamma rays give far less intense radiation, so the resting **O** states and their structures will not be sensitive to the gamma irradiation.

### The Calculated O State Fe_a3_^HS/IS/LS,3+^−H_2_O−Cu_B_^2+^ DNC Structures

Our calculated **O**[Fe_a3_^HS/IS/LS,3+^−H_2_O−Cu_B_^2+^] DNC structures are taken from our recent publication Ref. [32]. The initial geometries of the DNC model clusters were established based on the Cartesian coordinates of the *ba*_3_ C*c*O X-ray crystal structure 3S8G.^[25]^ Then geometry optimization calculations were performed using DFT broken-symmetry^[37–39]^/OLYP-D3(BJ)^[40]^/Triple-ξ-Polarization(TZP) plus COSMO^[17,18,41–44]^ solvation model methodology implemented within the ADF2017 software package.^[45–47]^ The inclusion of dispersion D3(BJ) force-field type effects on the geometries distinguishes our current Mossbauer calculations from those we performed previously both for the fit set of Fe structure complexes (see section ^57^Fe_a3_^3+^ Mössbauer Isomer Shift and Quadrupole Splitting Calculations), and for the **O** state structures. The inner cores of C(1 s), N(1 s), and O(1 s) were treated by frozen core approximation during geometry optimizations. Several Fe_a3_^HS/IS/LS,3+^−H_2_O−Cu_B_^2+^ local minima were found with the H_2_O molecule residing at different positions between Fe_a3_^3+^ and Cu_B_^2+^. Specifically, one structure for the Fe_a3_^LS,3+^−H_2_O−Cu_B_^2+^ state and four structures (noted as a, b, c, and d) for each of the Fe_a3_^HS,3+^−H_2_O−Cu_B_^2+^ and Fe_a3_^IS,3+^−H_2_O−Cu_B_^2+^ states were presented in our publication Ref. [32], and now are given in Table 2. For each broken-symmetry optimized geometry, we have also performed an Fe_a3_^3+^−Cu_B_^2+^ F-coupled single-point energy calculation and have presented the relative energies in Table 2.

**Figure 3.**
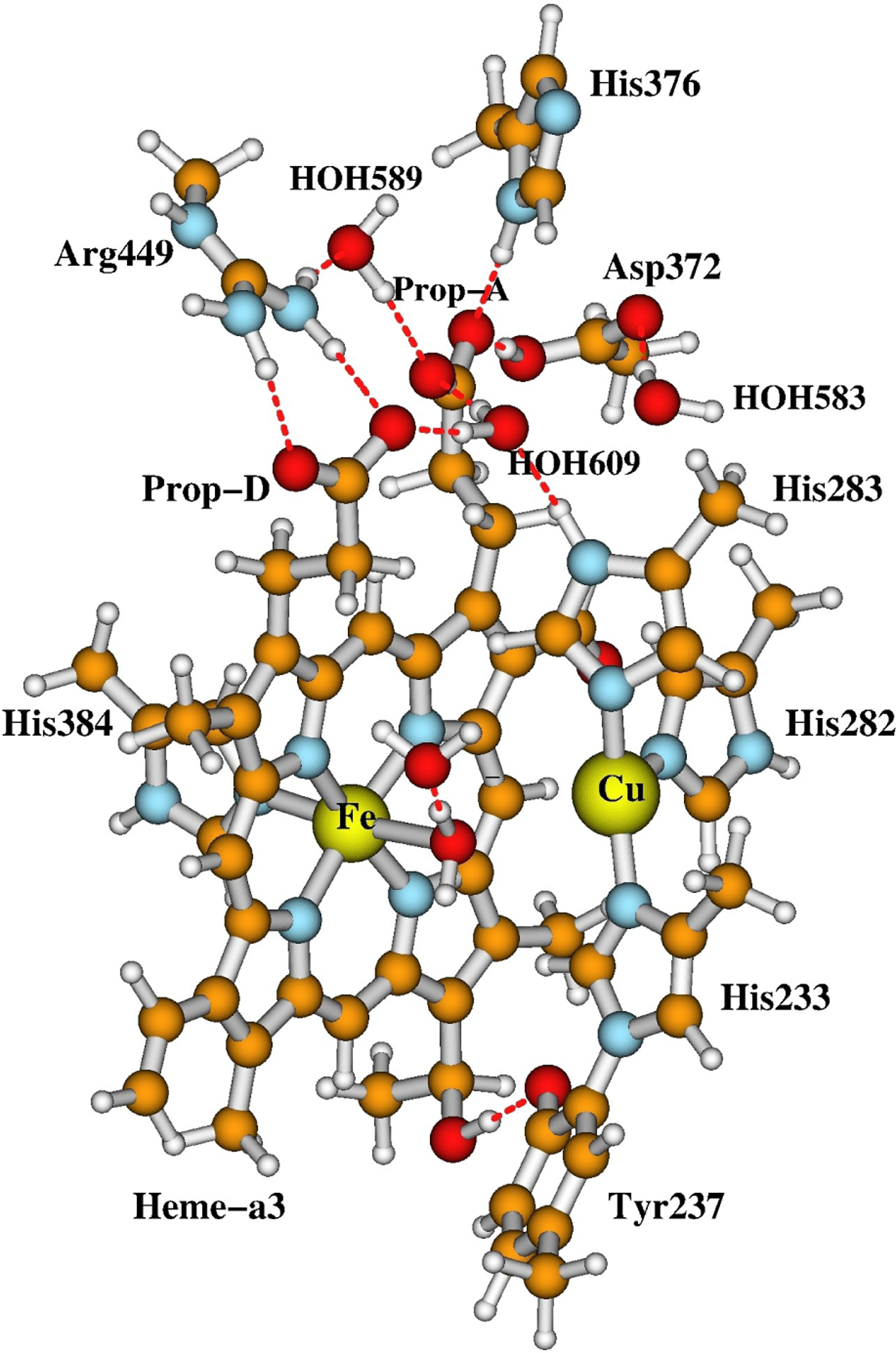
Our full DNC model cluster representing the Fe_a3_^HS,3+^−H_2_O−Cu_B_^2+^(a), Fe_a3_^IS,3+^−H_2_O−Cu_B_^2+^(a), and Fe_a3_^LS,3+^−H_2_O−Cu_B_^2+^ states, in which the H_2_O molecule is much closer to the Fe_a3_^3+^ site. The top and the central portions of the cluster are also shown in Figure 4. Linking hydrogen atoms were fixed during geometry optimization.

**Figure 4.**
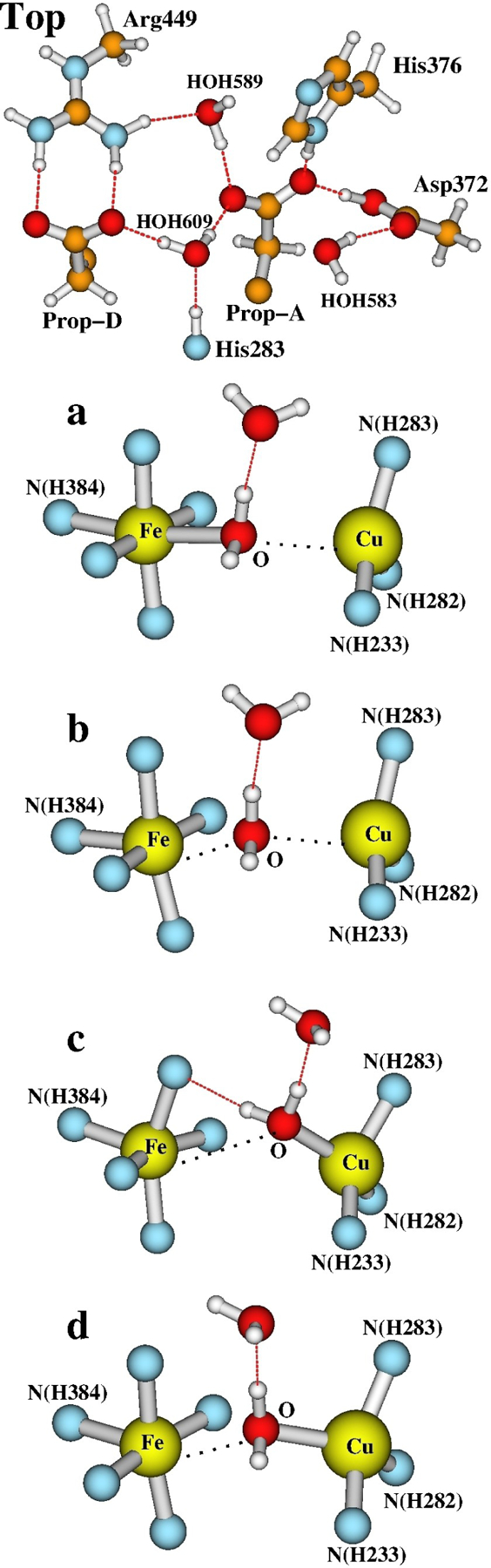
Four optimized **O** state DNC model clusters (noted as a, b, c, and d) for each of the Fe_a3_^HS,3+^−H_2_O−Cu_B_^2+^ and Fe_a3_^IS,3+^−H_2_O−Cu_B_^2+^ states and one structure for the Fe_a3_^LS,3+^−H_2_O−Cu_B_^2+^ state were presented in our publication Ref. [32]. Here is a closer look at the top and the central portions of these clusters. “a–d” show the central Fe_a3_^HS/IS,3+^−H_2_O−Cu_B_^2+^(a–d) structures. “a” also represents the central portion of the Fe_a3_^LS,3+^−H_2_O−Cu_B_^2+^ model cluster. The Fe−O, Cu−O and Fe−Cu distances of the optimized Fe_a3_^HS/IS,3+^−H_2_O−Cu_B_^2+^ (a–d) and Fe_a3_^LS,3+^−H_2_O−Cu_B_^2+^ structures are given in Table 2. Relative energies of different states are reported in Tables 2 and 3.

The four HS−Fe_a3_^3+^ structures Fe_a3_^HS,3+^−H_2_O−Cu_B_^2+^(a–d) are very similar to the corresponding IS Fe_a3_^3+^ structures Fe_a3_^IS,3+^−H_2_O−Cu_B_^2+^(a–d). Further, the optimized Fe_a3_^LS,3+^−H_2_O−Cu_B_^2+^ structure is also similar to Fe_a3_^HS/IS,3+^−H_2_O−Cu_B_^2+^(a) structures. The full model cluster representing the Fe_a3_^HS,3+^−H_2_O−Cu_B_^2+^(a), Fe_a3_^IS,3+^−H_2_O−Cu_B_^2+^(a), and the Fe_a3_^LS,3+^−H_2_O−Cu_B_^2+^ state is shown in Figure 3, in which the H_2_O molecule is much closer to the Fe_a3_^3+^ site with the distances (see Table 2) r(Fe_a3_^3+^−O)=2.39, 2.40, and 2.37 Å and r(Cu_B_^2+^−O)=2.94, 2.90, and 3.10 Å for the Fe_a3_^HS,3+^−H_2_O−Cu_B_^2+^(a), Fe_a3_^IS,3+^−H_2_O−Cu_B_^2+^(a), and Fe_a3_^LS,3+^−H_2_O−Cu_B_^2+^ structures, respectively. The H_2_O molecule is also H-bonding with another H_2_O molecule which probably originates from the Cu_B_-bound H_2_O ligand in the prior reaction cycle state **F** (see Figure 1). For a clearer view, the top and the central portions of this structure are also given in Figure 4. The central portions of the Fe_a3_^HS/IS,3+^−H_2_O−Cu_B_^2+^(b–c) are also shown in Figure 4.

### ^57^Fe_a3_^3+^ Mössbauer Isomer Shift and Quadrupole Splitting Calculations

In general, the isomer shifts (δ) can be calculated according to the fit equation [Eq. (1)]:

(1) δ=α[ρ(0)−A]+C

where ρ(0) is the calculated electron density at Fe nucleus and A is a predefined constant close to the value of ρ(0). The parameters α and *C* are normally obtained by linear fitting between the calculated ρ(0) and the experimental (exp) δ values of a set of Fe^2+^, Fe^3+^, and Fe^4+^ complexes. However, we have found that a global fitting of a single equation for all Fe^2+ ,3+,4+^ complexes in general underestimates the isomer shifts for the Fe^2+^ and Fe^4+^ sites, but overestimates the δ values for the Fe^3+^ state.^[48–50]^ In order to reasonably predict the ^57^Fe isomer shifts in different oxidation states, we have fit the parameters separately for the Fe^2+^ and for Fe^2.5+,3+,4+^ complexes with PW91, OLYP, and OPBE functionals,^[49,50]^ and have successfully predicted the isomer shifts for various states of the Fe−Fe, Fe−Mn, and Fe_4_S_4_ clusters in methane monooxygenase,^[49–51]^ ribonucleotide reductases,^[49,52–56]^ and APS-reductase.^[57]^ We have also calculated the isomer shift and quadrupole splitting values with OLYP functional for the Fe_a3_^2+/3+^ site of *ba*_3_ C*c*O based on several X-ray crystal structures.^[31]^ However, at that time, we did not propose that a single water molecule resides between the Fe_a3_^3+^ and Cu_B_^2+^ sites in the DNC of oxidized as-isolated C*c*O. In the current paper, we have performed the ρ(0) *vs*. δ_exp_ linear fitting for the OLYP-D3(BJ) potential on the same training set of the Fe^2.5+,3+,3.5+,4+^ complexes as we have done for the PW91, OLYP, and OPBE potentials.^[49,50]^ The training set contains 19 Fe^2.5+,3+,3.5+,4+^ sample complexes with total 30 Fe sites. Previously, we used our own program to calculate the electron density ρ(0) at the Fe nuclei. More recently, the ADF computer code package by default also reports the electron density at the nuclei. Based on their description, the electron density is not calculated exactly at the center of the nucleus. Instead, the electron density is calculated at sample points on a small spherical surface surrounding the center of a nucleus. The computed electron density in the output of ADF is the average electron density on these points. We now use that ρ(0) in ADF output to perform the linear fitting and the isomer shift calculations. We note that this procedure bears a close resemblance to the actual physical change in electron density-nuclear contact interaction (effectively over a thin spherical shell) when the ^57^Fe nucleus changes its radius and volume upon gamma ray excitation from the ground state (spin I=1/2) to its excited state (with spin I=3/2). This same excitation changes the shape of the ^57^Fe nucleus from spherical to ellipsoidal. That change produces the quadrupole splitting in the Mossbauer spectrum.

The details of the Fe^2.5+,3+,3.5+,4+^ complexes in the training set and the ρ(0) *vs*. δ_exp_ linear regression are given in the Supporting Information. Briefly, by taking the constant A = 11820.0, our linear fitting for the OLYP-D3(BJ) potential yields [Eq. (2)]:

(2) $$\delta \left({\text{Fe}}^{2.5+,3+,3.5+,4+}\right)=-0.337[\rho (0)-11820.0]+0.571\left(\text{mm}\;{\text{s}}^{-1}\right)$$

with correlation coefficient r = 0.946 and a standard deviation SD=0.068 mm s^−1^. We then performed single-point energy calculations on each of our optimized **O** state Fe_a3_^HS/IS/LS,3+^−H_2_O−Cu_B_^2+^ DNC model clusters in both broken-symmetry (representing Fe_a3_^3+^ Cu_B_^2+^ AF-coupled state) and F-coupled states with all electron and all TZP basis set to obtain the ρ(0) values and further predicted the ^57^Fe_a3_^3+^ isomer shift values based on Equation (2). The quadrupole splitting values (Δ*E*_Q_) were also obtained from ADF output in these calculations.

Normally the calculated electric field gradient (EFG) tensors *V* at the Fe nucleus are diagonalized and the eigenvalues are reordered so that |*V*_zz_| ≥ |*V*_yy_| ≥ |*V*_xx_|. The asymmetry parameter η is defined as [Eq. (3)]:

(3) $$\eta =\left(V_{\text{xx}}-V_{\text{yy}}\right)/V_{\text{zz}}$$

Then the Δ*E*_Q_ for ^57^Fe of the nuclear excited state (*I*=3/2) can be calculated as [Eq. (4)]:

(4) ΔEQ=(12)eQVzz(1+η2/3)12

where *e* is the electrical charge of a positive electron, *Q* is the nuclear quadrupole moment of Fe. Recently, the ADF software package determines the Δ*E*_Q_ value using *Q*=0.16 barn, the current best experimental value.

We have listed our calculated ^57^Fe_a3_^3+^ isomer shift and quadrupole splitting results for our **O** state Fe_a3_^HS/IS/LS,3+^−H_2_O−Cu_B_^2+^ DNC model clusters in Table 2.

## Results and Discussion

### Calculated Mössbauer Properties for the High-Spin−Fe_a3_^3+,HS^−H_2_O−Cu_B_^2+^(a–d) DNC Model Clusters

First, we compare our calculated Mössbauer properties with experimental data for Fe_a3_^3+^ in high-spin state. In Table 1, the observed isomer shift values for the Fe_a3_^HS,3+^ are around 0.41–0.48 mm s^−1^. Our calculated δ(Fe_a3_^3+,HS^) values (see Table 2) for our Fe_a3_^HS,3+^−H_2_O−Cu_B_^2+^(a–d) DNC models are 0.40–0.45 mm s^−1^, which are highly consistent with these experiments. The available observed Δ*E*_Q_(Fe_a3_^3+,HS^)_exp_ values for different C*c*Os are reported as 0.7, ~1, 1.0 ± 0.1, 1.10, and 1.3 mm s^−1^, while our calculated Δ*E*_Q_(Fe_a3_^3+,HS^) values are 0.77, 0.78, 0.80, 0.92, 1.21, and 1.24 mm s^−1^, which also match the experimental values very well.

Note that in our DNC models shown in Figures 3 and 4 and the corresponding calculation results in Table 2, there is another H_2_O molecule, which likely originates from the Cu_B_-bound H_2_O ligand in state **F** (see Figure 1), and has H-bonding interaction with the central H_2_O molecule. In the X-ray crystal structure 3S8G,^[25]^ a water molecule (HOH608) was seen 3.01 Å above one of the dioxygen atoms that is closer to Cu_B._. Therefore, the HOH608 may have H-bonding interaction with the H_2_O ligand in a position similar to Fe_a3_^3+^−H_2_O−Cu_B_^2+^(c) (Figure 4c). No other H_2_O molecules were identified within the H-bonding distances around the dioxygen in 3S8G. This probably implies that not all C*c*O molecules in the crystal have an H-bonding H_2_O interacting with the central bridging H_2_O molecule, and even if there is an H-bonding H_2_O molecule in some of the C*c*O DNCs, the H-bonding patterns and the positions of the H-bonding H_2_O molecules may differ, and therefore, they may not be identified in the X-ray crystal structure.

Since it is not clear whether there is an H-bonding H_2_O molecule in the DNC of the **O** state, in our previous work in Ref. [32], we also performed broken-symmetry state geometry optimizations on the four Fe_a3_^HS,3+^−H_2_O−Cu_B_^2+^(a)-(d) structures obtained by removing the H-bonding H_2_O. The four corresponding optimized structures (S) were labeled as S1, S2, S3, and S4 in Figure 6 of Ref. [32], respectively. Their main bond distances and the calculated energies were given in Table 2 of Ref. [32], and are also given in Table 3 here, together with the single-point energy Fe_a3_^HS,3+^−Cu_B_^2+^ F-coupled state calculations on the broken-symmetry optimized geometries.

Without the H-bonding H_2_O molecule, the binding of the H_2_O ligand with the two metal sites, especially with the Fe_a3_^HS,3+^ site, are weakened. But still the overlap of the S1–S4 structures with the DNC of 3S8G shows the H_2_O molecules in S1–S4 reside along the apparent dioxygen species direction in 3S8G (see Figure 2). We therefore also calculated the ^57^Fe_a3_^HS,3+^ Mössbauer isomer shift and quadrupole splitting properties of these four structures in both AF- (broken-symmetry) and F-coupled states, and presented the results in Table 3. The calculated isomer shifts are almost the same as those given in Table 2, and are again highly consistent with the observed values.

**Figure 5.**
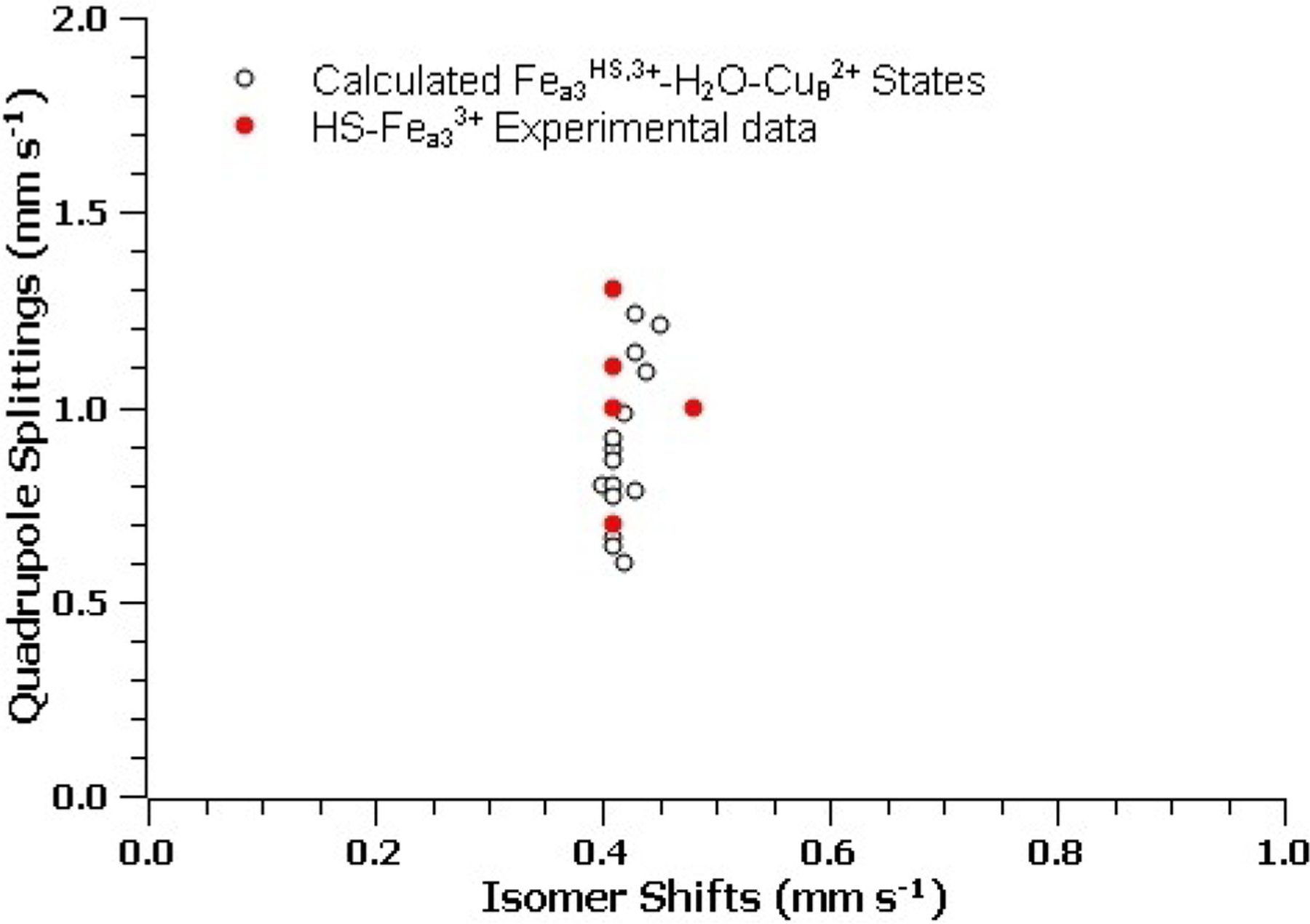
Calculated (black circles, data are given in Tables 2 and 3) *versus* several experimentally defined (red dots, data are given in Table 1) Mössbauer isomer shift and quadrupole splitting values for the high-spin (HS) ^57^Fe_a3_^3+^ site in C*c*Os.

Based on the calculated ^57^Fe_a3_^HS,3+^ Mössbauer properties given in Tables 2 and 3, it appears that the ^57^Fe_a3_^HS,3+^ isomer shifts only slightly vary with the position of the H_2_O molecule, whether or not there is an H-bonding H_2_O molecule, and whether Fe_a3_^HS,3+^ is F- or AF-coupled with Cu_B_^2+^. However, the ^57^Fe_a3_^HS,3+^ quadrupole splitting values are very sensitive to these factors. This explains that the observed Mössbauer spectra are broad, that the parameters are difficult to define precisely, and that many of the reported parameters are averaged values.^[33–35]^ Our calculated *versus* the several reported experimentally defined ^57^Fe_a3_^HS,3+^ Mössbauer isomer shift and quadrupole splitting values are compared in Figure 5.

In general, when the H_2_O molecule is much closer to the Fe_a3_^HS,3+^ site (Fe_a3_^HS,3+^−H_2_O−Cu_B_^2+^(a)-(b)), the calculated ^57^Fe_a3_^HS,3+^ quadrupole splitting values for the F- and AF-coupled states are similar to each other. On the other hand, when the H_2_O molecule is much closer to the Cu_B_^2+^ site (Fe_a3_^HS,3+^−H_2_O−Cu_B_^2+^(c)-(d)), the calculated Δ*E*_Q_(Fe_a3_^HS,3+^) values for the F-coupled state are much larger than the corresponding AF-coupled state. The observed Δ*E*_Q_(Fe_a3_^HS,3+^)_exp_ values around 1.0 to 1.3 mm s^−1^ (see Table 1)^[33–36]^ likely arise from the DNC structures in which the H_2_O molecule is much closer to the Cu_B_^2+^ site and the Fe_a3_^HS,3+^ and Cu_B_^2+^ sites are F-coupled (see Tables 2 and 3). Meanwhile, the observed Δ*E*_Q_(Fe_a3_^HS,3+^)_exp_ values around 0.7 mm s^−1^ probably come from the structures where the H_2_O molecule is close to the Fe_a3_^HS,3+^ site (whether the Fe_a3_^HS,3+^ and Cu_B_^2+^ sites are F- or AF-coupled), or from the structures where the H_2_O molecule is much closer to Cu_B_^2+^ and the two metal sites are AF-coupled.

### Calculated Mössbauer Properties for the Intermediate- and Low-Spin−Fe_a3_^IS/LS,3+^−H_2_O−Cu_B_^2+^ DNC Model Clusters

In *Tt c*_1_*aa*_3_ and *ba*_3_ at certain pH or at low temperature (see Table 1),^[35,36]^ a “low-spin” Fe_a3_^3+^ species was observed with δ= 0.29 mm s^−1^ and Δ*E*_Q_=2.21/2.24 mm s^−1^. Although this was proposed to be a low-spin Fe_a3_^3+^ species, such δ and Δ*E*_Q_ values could also originate from an intermediate-spin Fe^3+^ site. Therefore, we have performed Mössbauer property calculations on our IS and LS Fe_a3_^IS/LS,3+^−H_2_O−Cu_B_^2+^ models listed in Table 2.

Unlike the high-spin Fe_a3_^HS,3+^−H_2_O−Cu_B_^2+^ models, the F- and AF-coupled spin states yield essentially the same isomer shift and quadrupole splitting results for the same Fe_a3_^IS/LS,3+^−H_2_O−Cu_B_^2+^ structure. Further, the position of the H_2_O molecule does not have significant effect on the calculated ^57^Fe_a3_^IS,3+^ isomer shift and quadrupole splitting values on the four Fe_a3_^IS,3+^−H_2_O−Cu_B_^2+^(a)-(d) structures. Overall, the calculated δ(Fe_a3_^IS,3+^) only varies from 0.34 to 0.36 mm s^−1^, and Δ*E*_Q_(Fe_a3_^IS,3+^) from 2.33 to 2.66 mm s^−1^.

For the low-spin Fe_a3_^LS,3+^−H_2_O−Cu_B_^2+^ structure, our calculations give δ(Fe_a3_^LS,3+^)=0.31 mm s^−1^ and Δ*E*_Q_(Fe_a3_^LS,3+^)= 2.96 mm s^−1^.

Compared with experimental δ=0.29 mm s^−1^ and Δ*E*_Q_= 2.21/2.24 mm s^−1^ values, the calculated δ(Fe_a3_^LS,3+^)=0.31 mm s^−1^ is a little closer to experiment than the calculated δ(Fe_a3_^IS,3+^) values (0.34 to 0.36 mm s^−1^). However, the Δ*E*_Q_(Fe_a3_^LS,3+^)= 2.96 mm s^−1^ deviates more from experiment than the Δ*E*_Q_(Fe_a3_^IS,3+^) results (2.33 to 2.66) mm s^−1^. In the Supporting Information, we have used linear regression on the training set of Fe complexes to find the standard deviation (SD) of the fit for the isomer shifts, SD=0.068 mm s^−1^, and also the standard deviation of the fit for the corresponding quadrupole splittings, SD=0.30 mm s^−1^. The experimentally observed Mössbauer spectrum with δ=0.29 mm s^−1^ and Δ*E*_Q_=2.21/2.24 mm s^−1^ spectra has an isomer shift within 1 SD from either the DFT calculated low-spin or an intermediate-spin Fe_a3_^3+^ species. By contrast, for the predicted versus experimental quadrupole splitting, the calculated low-spin quadrupole splitting differs by more than 2 SD from experiment, while the calculated intermediate-spin quadrupole splittings are much closer < 1.5 SD.

In addition, the intermediate-spin Fe_a3_^IS,3+^−H_2_O−Cu_B_^2+^(a)-(d) structures have lower energy than the low-spin Fe_a3_^LS,3+^−H_2_O−Cu_B_^2+^ state, and the structure Fe_a3_^IS,3+^−H_2_O−Cu_B_^2+^(c) has calculated δ=0.34 mm s^−1^ and Δ*E*_Q_=2.33 mm s^−1^, which are the closest to the experiment. Therefore, the experimentally observed δ=0.29 mm s^−1^ and Δ*E*_Q_=2.21/2.24 mm s^−1^ spectra are probably from intermediate-spin Fe_a3_^3+^ DNCs.

## Conclusions

In our previous study,^[32]^ we proposed that a single water molecule is in between the Fe_a3_^3+^ and Cu_B_^2+^ sites in the resting oxidized as-isolated **O** state of C*c*O. Depending on the Fe_a3_^3+^ Cu_B_^2+^ distance and presence or absence of H-bonding to another H_2_O molecule, this single H_2_O molecule can coordinate to either the Fe_a3_^3+^ or the Cu_B_^2+^ site, or can reside at different positions between the Fe_a3_^3+^ and Cu_B_^2+^ sites that are energetically very close on the potential energy surface. We therefore have also proposed that the extended peroxide type electron density between Fe_a3_^3+^ and Cu_B_^2+^ observed in several C*c*O X-ray crystal structures^[23–25]^ results are the consequence of the superposition of the electron density of a water molecule at different locations between Fe_a3_^3+^ and Cu_B_^2+^ in different C*c*O molecules within the crystals.

The structural heterogeneities of the DNC in the resting oxidized state C*c*Os (**O** state) were demonstrated by earlier ^57^Fe^3+^ Mössbauer experiments,^[33–36]^ in which the spectra were broad, the parameters were difficult to define precisely, and the reported parameters were averaged values.

In this paper, we have calculated the ^57^Fe_a3_^3+^ Mössbauer isomer shift (δ) and quadrupole splitting (Δ*E*_Q_) properties of the resting state **O**[Fe_a3_^3+^−H_2_O−Cu_B_^2+^] DNC structures that we obtained in Ref. [32], and have compared the calculated results with the available experimental values (see Table 1). Overall, the span of our calculated δ and Δ*E*_Q_ results for the high-spin Fe_a3_^3+^ DNC structures agree very well with the experimental values. Our calculations show that the change of the high-spin-^57^Fe_a3_^3+^ isomer shift among different DNC structures is within 0.05 mm s^−1^. However, the quadrupole splitting values vary with the position of the central H_2_O molecule, whether or not this H_2_O molecule has H-bonding interaction with another H_2_O molecule, and whether the high-spin Fe_a3_^3+^ site is F- or AF-coupled with Cu_B_^2+^. The observed Δ*E*_Q_ values around 1.0 to 1.3 mm s^−1^ (see Table 1)^[33–36]^ likely result from the DNC structures in which the central H_2_O molecule is much closer to the Cu_B_^2+^ site and the high-spin Fe_a3_^3+^ and Cu_B_^2+^ sites are Fcoupled. Meanwhile the observed Δ*E*_Q_ values around 0.7 mm s^−1^ probably result from the structures where the H_2_O molecule is close to the high-spin Fe_a3_^3+^ site with the two metal sites either F- or AF-coupled, or from the structures where the H_2_O molecule is much closer to Cu_B_^2+^ and the two metal sites are AF-coupled.

Our calculations also show that the observed “low-spin” Fe_a3_^3+^ species with δ=0.29 mm s^−1^ and Δ*E*_Q_=2.21/2.24 mm s^−1^ more probably arises from an intermediate-spin−Fe_a3_^3+^ state, which exists at low temperature^[36]^ or is more populated at certain pH values.^[35]^

Overall, our calculations demonstrate that the structural heterogeneities of the resting as-isolated oxidized state observed in several Mössbauer properties experiments are very consistent with the DFT predicted properties and structures of a single H_2_O molecule bridging the Fe_a3_^3+^ and Cu_B_^2+^ sites with variable positions, with further variations explained by the Fe_a3_^3+^ spin states, and by the different spin-couplings between Fe_a3_^3+^ and Cu_B_^2+^.

## Supplementary Material

ChemPhysChem Supporting Information

## Figures and Tables

**Table 1.** Experimentally observed^[33–36] 57^Fe_a3_^3+^ Mössbauer isomer shifts (δ, mm s^−1^) and quadrupole splittings (Δ*E*_Q_, mm s^−1^) in different resting oxidized cytochrome oxidases.

Oxidases	Ref.	Fe_a3_^3+^-Spin^[a]^	*T* [K]	δ	Δ*E*_Q_	Note
*Tt c*_1_ *aa*_3_	[33]	HS	4.2	0.41	1.10	The parameters given are averaged values. The spectrum is broad, suggesting heterogeneities.
bovine aa_3_	[34]	HS	4.2	0.48 ± 0.06	1.0 ± 0.1	The *a*_3_ sites also appear to be heterogeneous. The parameters reported are also averaged values.
*Tt c*_1_ *aa*_3_	[35]	HS	4.2	0.41	1.3	Only one set of the HS parameters was defined. However, at least 3 and 2 HS *a*_3_ species were observed at pH = 5.7 and pH = (6.5, 7.8, 9.3), respectively. And the LS *a*_3_ species was found at pH = 5.7, 7.8, and 9.3.
		LS ^b^	4.2	0.29	2.21	
*Tt ba*_3_	[36]	HS	4.2–245	0.41	0.7	The first two HS and LS species coexist at 4.2 K. However, the LS component changes to a different HS species with Δ*E*_Q_ ≈ 1 mms^−1^ when *T>* 190 K, and the transition is complete at 245 K.
		LS ^[b]^	4.2–190	0.29	2.24	
		HS	> 190	0.41 ^[c]^	~1	

[a] HS stands for high-spin; IS is for intermediate-spin; and LS is for low-spin.

[b] Although it was suggested as LS−Fe_a3_^3+^, it is not certain whether it is LS or IS Fe_a3_^3+^ site.

[c] This δ value was not specifically reported in the paper.^[36]^ However, from the context, we assume it is the same as another HS species, 0.41 mm s^−1^.

**Table 2.** OLYP-D3-BJ calculated distances (in Å), relative energies (Δ*E*, in kcal mol^−1^), Mössbauer isomer shift (δ, in mm s^−1^) and quadrupole splitting (Δ*E*_Q_, in mm s^−1^) values of the resting as-isolated Fe_a3_^3+^−H_2_O−Cu_B_^2+^ optimized DNC structures with different Fe_a3_^3+^−Cu_B_^2+^ spin states.

Fe_a3_^3+^-Spin^[a]^	Structure	Distances [Å] Distances			Spin-Coupling	Δ*E*	δ	Δ*E*_Q_
		Fe−O	Cu−O	Fe ⋯ Cu				
HS	Fe_a3_^HS,3^+−H_2_O−Cu_B_^2+^(a)	2.39	2.94	4.98	AF	0.0	0.40	0.80
					F	0.0	0.41	0.80
	Fe_a3_^HS,3+^−H_2_O−Cu_B_^2+^(b)	2.47	2.77	4.90	AF	−0.2	0.41	0.92
					F	−0.2	0.41	0.80
	Fe_a3_^HS,3+^-H_2_O−Cu_B_^2+^(c)	3.55	2.21	4.73	AF	0.3	0.43	0.78
					F	−0.4	0.45	1.21
	Fe_a3_^HS,3+^−H_2_O−Cu_B_^2+^(d)	3.26	2.20	4.73	AF	0.5	0.41	0.77
					F	0.3	0.43	1.24
IS	Fe_a3_^IS,3+^−H_2_O−Cu_B_^2+^(a)	2.40	2.90	4.94	AF	−6.2	0.36	2.42
					F	−6.2	0.36	2.42
	Fe_a3_^IS,3+^−H_2_O−Cu_B_^2+^(b)	2.46	2.67	4.78	AF	−6.5	0.36	2.50
					F	−6.5	0.36	2.50
	Fe_a3_^IS,3+^−H_2_O−Cu_B_^2+^(c)	3.44	2.26	4.71	AF	−4.3	0.34	2.34
					F	−4.1	0.34	2.33
	Fe_a3_^IS,3+^−H_2_O−Cu_B_^2+^(d)	3.00	2.24	4.56	AF	−5.5	0.35	2.66
					F	−5.5	0.35	2.65
LS	Fe_a3_^LS,3+^−H_2_O−Cu_B_^2+^	2.37	3.10	5.04	AF	−2.7	0.31	2.96
					F	−2.7	0.31	2.96

[a] HS stands for high-spin; IS is for intermediate-spin; and LS is for low-spin.

**Table 3.** OLYP-D3-BJ calculated properties (relative energies Δ*E* in kcal mol^−1^, Mössbauer isomer shift δ in mm s^−1^, quadrupole splitting Δ*E*_Q_ in mm s^−1^) for the four geometry optimized high-spin−Fe_a3_^3+^ DNC structures starting from Fe_a3_^3+,HS^−H_2_O−Cu_B_^2+^(a–d) by deleting the H_2_O molecule which has H-bonding interaction with the central H_2_O molecule.^[a]^

Starting from Structure	Name in Ref. [32]	Optimized Geometry [Å]			Spin-Coupling	Δ*E*	δ	Δ*E*_Q_
		Fe−O	Cu−O	Fe ⋯ Cu				
Fe_a3_^HS,3+^−H_2_O−Cu_B_^2+^(a)	S1	2.61	2.82	5.03	AF	0.0	0.41	0.85
					F	0.0	0.41	0.86
Fe_a3_^HS,3+^−H_2_O−Cu_B_^2+^(b)	S2	2.97	2.52	4.98	AF	−1.1	0.41	0.66
					F	−1.2	0.42	0.98
Fe_a3_^HS,3+^−H_2_O−Cu_B_^2+^(c)	S3	3.54	2.29	4.76	AF	−1.5	0.42	0.60
					F	−1.9	0.44	1.09
Fe_a3_^HS,3+^−H_2_O−Cu_B_^2+^(d)	S4	3.07	2.41	4.87	AF	−0.8	0.41	0.64
					F	−1.1	0.43	1.14

[a]. These four geometry optimized structures (S) were given as S1, S2, S3, and S4, respectively, in Table 2 of Ref. [32].

## Data Availability

The data that support the findings of this study are available in the supplementary material of this article.

## References

[1] Richter OMH, Ludwig B, Rev. Physiol. Biochem. Pharmacol 2003, 147, 47–74. 12783267 10.1007/s10254-003-0006-0

[2] Babcock GT, Wikström M, Nature 1992, 356, 301–309. 1312679 10.1038/356301a0

[3] Ferguson-Miller S, Babcock GT, Chem. Rev 1996, 96, 2889–2907. 11848844 10.1021/cr950051s

[4] Wikström M, Biochim. Biophys. Acta 2012, 1817, 468–475. 22079200 10.1016/j.bbabio.2011.10.010

[5] Kaila VRI, Verkhovsky MI, Wikström M, Chem. Rev 2010, 110, 7062–7081. 21053971 10.1021/cr1002003

[6] Konstantinov AA, FEBS Lett. 2012, 586, 630–639. 21889506 10.1016/j.febslet.2011.08.037

[7] von Ballmoos C, Adelroth P, Gennis RB, Brzezinski P, Biochim. Biophys. Acta Bioenerg 2012, 1817, 650–657. 10.1016/j.bbabio.2011.11.015 22172736

[8] Wikström M, Krab K, Sharma V, Chem. Rev 2018, 118, 2469–2490. 29350917 10.1021/acs.chemrev.7b00664 PMC6203177

[9] Blomberg MRA, Front. Chem 2021, 9, 640155. 33937193 10.3389/fchem.2021.640155 PMC8079940

[10] Yoshikawa S, Shimada A, Chem. Rev 2015, 115, 1936–1989. 25603498 10.1021/cr500266a

[11] Ishigami I, Hikita M, Egawa T, Yeh SR, Rousseau DL, Biochim. Biophys. Acta Bioenerg 2015, 1847, 98–108. 10.1016/j.bbabio.2014.09.008 PMC4254173 25268561

[12] Blomberg MRA, Siegbahn PEM, Biochim. Biophys. Acta Bioenerg 2015, 1847, 364–376. 10.1016/j.bbabio.2014.12.005 25529353

[13] Blomberg MRA, Siegbahn PEM, Biochim. Biophys. Acta Bioenerg 2015, 1847, 1173–1180. 10.1016/j.bbabio.2015.06.008 26072193

[14] Blomberg MRA, Siegbahn PEM, Biochim. Biophys. Acta 2012, 1817, 495–505. 21978537 10.1016/j.bbabio.2011.09.014

[15] Noodleman L, Han Du W-G, Fee JA, Götz AW, Walker RC, Inorg. Chem 2014, 53, 6458–6472. 24960612 10.1021/ic500363h PMC4095914

[16] Han Du W-G, Götz AW, Yang LH, Walker RC, Noodleman L, Phys. Chem. Chem. Phys 2016, 18, 21162–21171. 27094074 10.1039/c6cp00349d PMC4972664

[17] Han Du W-G, Götz AW, Noodleman L, Inorg. Chem 2018, 57, 1048–1059. 29308889 10.1021/acs.inorgchem.7b02461 PMC5825212

[18] Han Du W-G, Götz AW, Noodleman L, Inorg. Chem 2019, 58, 13933–13944. 31566371 10.1021/acs.inorgchem.9b01840 PMC6839913

[19] Noodleman L, Han Du W-G, McRee D, Chen Y, Goh T, Götz AW, Phys. Chem. Chem. Phys 2020, 22, 26652–26668. 33231596 10.1039/d0cp04848h PMC7727307

[20] Moody AJ, Biochim. Biophys. Acta Bioenerg 1996, 1276, 6–20. 10.1016/0005-2728(96)00035-7 8764888

[21] Ostermeier C, Harrenga A, Ermler U, Michel H, Proc. Natl. Acad. Sci. USA 1997, 94, 10547–10553. 9380672 10.1073/pnas.94.20.10547 PMC23397

[22] Qin L, Hiser C, Mulichak A, Garavito RM, Ferguson-Miller S, Proc. Natl. Acad. Sci. USA 2006, 103, 16117–16122. 17050688 10.1073/pnas.0606149103 PMC1616942

[23] Koepke J, Olkhova E, Angerer H, Muller H, Peng GH, Michel H, Biochim. Biophys. Acta 2009, 1787, 635–645. 19374884 10.1016/j.bbabio.2009.04.003

[24] Aoyama H, Muramoto K, Shinzawa-Itoh K, Hirata K, Yamashita E, Tsukihara T, Ogura T, Yoshikawa S, Proc. Natl. Acad. Sci. USA 2009, 106, 2165–2169. 19164527 10.1073/pnas.0806391106 PMC2650126

[25] Tiefenbrunn T, Liu W, Chen Y, Katritch V, Stout CD, Fee JA, Cherezov V, PLoS One 2011, 6, e22348. 21814577 10.1371/journal.pone.0022348 PMC3141039

[26] Hirata K, Shinzawa-Itoh K, Yano N, Takemura S, Kato K, Hatanaka M, Muramoto K, Kawahara T, Tsukihara T, Yamashita E, Tono K, Ueno G, Hikima T, Murakami H, Inubushi Y, Yabashi M, Ishikawa T, Yamamoto M, Ogura T, Sugimoto H, Shen JR, Yoshikawa S, Ago H, Nat. Methods 2014, 11, 734–U174. 24813624 10.1038/nmeth.2962

[27] Andersson R, Safari C, Dods R, Nango E, Tanaka R, Yamashita A, Nakane T, Tono K, Joti Y, Bath P, Dunevall E, Bosman R, Nureki O, Iwata S, Neutze R, Branden G, Sci. Rep 2017, 7, 4518. 28674417 10.1038/s41598-017-04817-z PMC5495810

[28] Ueno G, Shimada A, Yamashita E, Hasegawa K, Kumasaka T, Shinzawa-Itoh K, Yoshikawa S, Tsukihara T, Yamamoto M, J. Synchrotron Radiat 2019, 26, 912–921. 31274413 10.1107/S1600577519006805 PMC6613116

[29] Kolbe F, Safarian S, Piorek Z, Welsch S, Muller H, Michel H, Nat. Commun 2021, 12, 6903. 34824221 10.1038/s41467-021-27174-y PMC8617209

[30] Han Du W-G, Noodleman L, Inorg. Chem 2013, 52, 14072–14088. 24262070 10.1021/ic401858s PMC3925067

[31] Han Du W-G, Noodleman L, Inorg. Chem 2015, 54, 7272–7290. 26192749 10.1021/acs.inorgchem.5b00700 PMC4525772

[32] Han Du W-G, McRee D, Götz AW, Noodleman L, Inorg. Chem 2020, 59, 8906–8915. 32525689 10.1021/acs.inorgchem.0c00724 PMC8114904

[33] Kent TA, Munck E, Dunham WR, Filter WF, Findling KL, Yoshida T, Fee JA, J. Biol. Chem 1982, 257, 12489–12492. 6290469

[34] Kent TA, Young LJ, Palmer G, Fee JA, Munck E, J. Biol. Chem 1983, 258, 8543–8546. 6305992

[35] Rusnak FM, Munck E, Nitsche CI, Zimmermann BH, Fee JA, J. Biol. Chem 1987, 262, 16328–16332. 2824489

[36] Zimmermann BH, Nitsche CI, Fee JA, Rusnak F, Munck E, Proc. Natl. Acad. Sci. USA 1988, 85, 5779–5783. 2842747 10.1073/pnas.85.16.5779 PMC281848

[37] Noodleman L, J. Chem. Phys 1981, 74, 5737–5743.

[38] Noodleman L, Case DA, Adv. Inorg. Chem 1992, 38, 423–470.

[39] Noodleman L, Lovell T, Han W-G, Liu T, Torres RA, Himo F, in: Comprehensive Coordination Chemistry II, From Biology to Nanotechnology, ed. Lever AB, Elsevier Ltd, 2003, vol. 2, pp. 491–510.

[40] Grimme S, Ehrlich S, Goerigk L, J. Comput. Chem 2011, 32, 1456–1465. 21370243 10.1002/jcc.21759

[41] Klamt, Schüürmann G, J. Chem. Soc. Perkin Trans 2 1993, 799–805.

[42] Klamt A, J. Phys. Chem 1995, 99, 2224–2235.

[43] Klamt A, Jonas V, J. Chem. Phys 1996, 105, 9972–9981.

[44] Pye CC, Ziegler T, Theor. Chem. Acc 1999, 101, 396–408.

[45] ADF, Amsterdam Density Functional Software, SCM, Theoretical Chemistry, Vrije Universiteit, Amsterdam, The Netherlands. http://www.scm.com.

[46] te Velde G, Bickelhaupt FM, Baerends EJ, Guerra CF, Van Gisbergen SJA, Snijders JG, Ziegler T, J. Comput. Chem 2001, 22, 931–967.

[47] Guerra CF, Visser O, Snijders JG, te Velde G, Baerends EJ, in: Methods and techniques for computational chemistry, eds. Clementi E, Corongiu C, STEF, Cagliari, 1995, pp. 303–395.

[48] Liu T, Lovell T, Han W-G, Noodleman L, Inorg. Chem 2004, 43, 6858–6858. 10.1021/ic0206443 14731023

[49] Han W-G, Liu T, Lovell T, Noodleman L, J. Comput. Chem 2006, 27, 1292–1306. 16786546 10.1002/jcc.20402

[50] Han W-G, Noodleman L, Inorg. Chim. Acta 2008, 361, 973–986. 10.1016/j.ica.2007.06.007 PMC2391136 19262682

[51] Han W-G, Noodleman L, Inorg. Chem 2008, 47, 2975–2986. 18366153 10.1021/ic701194b

[52] Han W-G, Noodleman L, Dalton Trans. 2009, 6045–6057. 19623405 10.1039/b903847g PMC2746754

[53] Han W-G, Giammona DA, Bashford D, Noodleman L, Inorg. Chem 2010, 49, 7266–7281. 20604534 10.1021/ic902051t PMC2919573

[54] Han W-G, Noodleman L, Theor. Chem. Acc 2010, 125, 305–317. 20445806 10.1007/s00214-009-0566-4 PMC2863024

[55] Han W-G, Noodleman L, Inorg. Chem 2011, 50, 2302–2320. 21322584 10.1021/ic1020127 PMC3059405

[56] Han W-G, Sandala GM, Giammona DA, Bashford D, Noodleman L, Dalton Trans. 2011, 40, 11164–11175. 21837345 10.1039/c1dt10950b PMC3604995

[57] Bhave DP, Han W-G, Pazicni S, Penner Hahn JE, Carroll KS, Noodleman L, Inorg. Chem 2011, 50, 6610–6625. 21678934 10.1021/ic200446c PMC3134165

