# Supplementary material for: DFT Calculations for Mössbauer Properties on Dinuclear Center Models of the Resting Oxidized Cytochrome c Oxidase
Source: Chemphyschem. Author manuscript; Available in PMC 2022 Apr 29. (PMC9054037; doi:10.1002/cphc.202100831)
Supplement: ChemPhysChem Supporting Information [file NIHMS1799927-supplement-ChemPhysChem_Supporting_Information.pdf]

ChemPhysChem

Supporting Information

DFT Calculations for Mössbauer Properties on Dinuclear Center Models of the Resting Oxidized Cytochrome *c* Oxidase

Wen-Ge Han Du, Andreas W. Götz, and Louis Noodleman*

1. Linear Fitting for $^{57}Fe^{3+}$ Isomer Shifts Using OLYP-D3(BJ) Functional

The isomer shift is proportional to the electron density ($\rho(0)$) difference at the Fe nuclei between the studied and a reference system (normally α-Fe at 300 K), and can be described as

$$\delta = \alpha[\rho(0) - A] + C \qquad \text{(S1)}$$

where A is a constant chosen close to the electron density at the Fe nucleus in the reference state. Here we choose A = 11820.0. $\rho(0)$ is calculated for a set of Fe complexes (whose isomer shifts (δ_{exp}) are known from experiment) from density functional theory, and α and C can be obtained by a linear regression between the calculated $\rho(0)$ and δ_{exp}. The isomer shifts of other Fe compounds then can be predicted.

The detailed fitting procedure and the sample 19 $Fe^{2.5+,3+,3.5+,4+}$ complexes and relevant literature citations were given in reference 1. These complexes (totally 30 Fe sites) are also given in **Table S1**. Previously, we used our own program to calculate the electron density $\rho(0)$ at the Fe nuclei. Recently, ADF by default also reports the electron density at the nuclei. Based on their description, the electron density is not calculated exactly at the center of the nucleus, however, at points on a small spherical surface around the center of a nucleus. The printed electron density in the output of ADF is the average electron density on these points. We now use the $\rho(0)$ in ADF output to perform the linear fitting and the isomer shift calculations. We have reoptimized the geometries and calculated the $\rho(0)$ values of the 19 $Fe^{2.5+,3+,3.5+,4+}$ complexes using the OLYP-D3(BJ) functional with the all-electron triple-ζ plus polarization (TZP) Slater type basis set within the COSMO solvation model in ADF2017 with a reasonable polar environment $\varepsilon = 32.6$ (the dielectric constant for methanol).

The experimental Fe isomer shifts (δ_T) were taken at different temperatures (T). We therefore shift them to a common temperature of 4.2 K, by taking account of the second-order Doppler effect. The offset given by ($\delta_{4.2K} - \delta_{300K}$) for this correction is approximately 0.12 mm s^{-1}, and this is expected to be linear with temperature.[1,2] The OLYP-D3(BJ) calculated $\rho(0)$, the experimental temperature, the measured δ_T, the shifted $\delta_{4.2K}$ for our $Fe^{2.5+,3+,3.5+,4+}$ training set, and the relevant references are all given in **Table S1**. Based on the correlation

$$\delta_{4.2K} = \alpha[\rho(0) - 11820.0] + C \qquad \text{(S2)}$$

The new linear regression (see **Figure S1**, totally N = 30 Fe sites) yields $\alpha = -0.337$ and $C = 0.571$ mm s^{-1}. The correlation coefficient is $r = -0.946$ with a standard deviation SD = 0.068 mm s^{-1}.

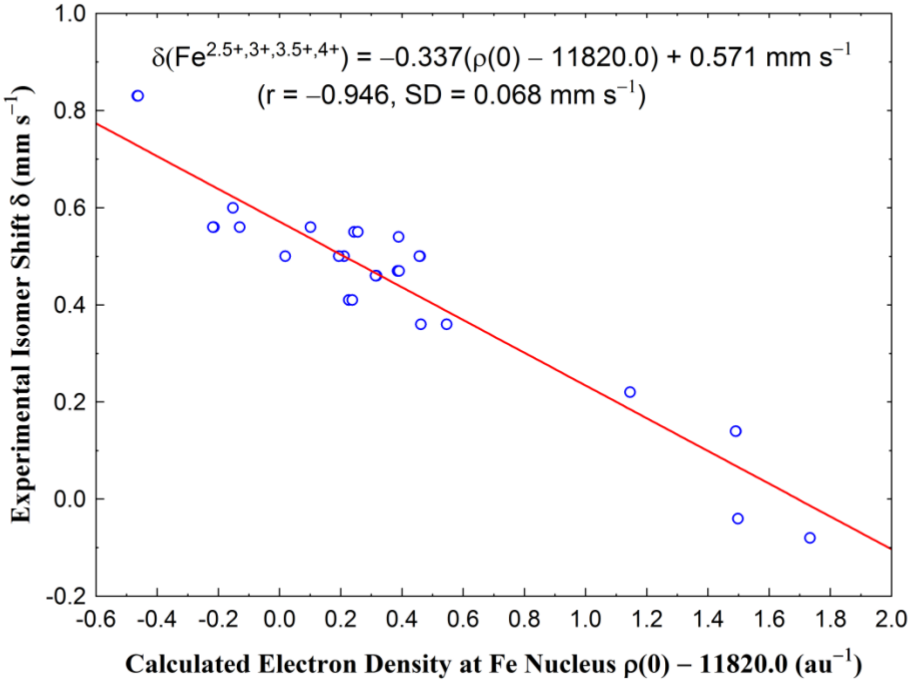

Figure S1. Correlations between the OLYP-D3(BJ) calculated electron densities at Fe nuclei and the experimental isomer shifts ($\delta_{4.2K}$) for 19 $Fe^{2.5+,3+,3.5+,4+}$ (totally 30 Fe sites) sample complexes.

Using the new fitted parameters α and C, we calculated the isomer shifts (δ_{cal}) for these 19 $Fe^{2.5+,3+,3.5+,4+}$ complexes and also gave the δ_{cal} values in **Table S1**. Further, we performed the linear regression between the calculated and experimental isomer shifts for these sample Fe complexes. The correlation plot of $\delta_{4.2K} = A\delta_{cal} + B$ is shown in **Figure S2**.

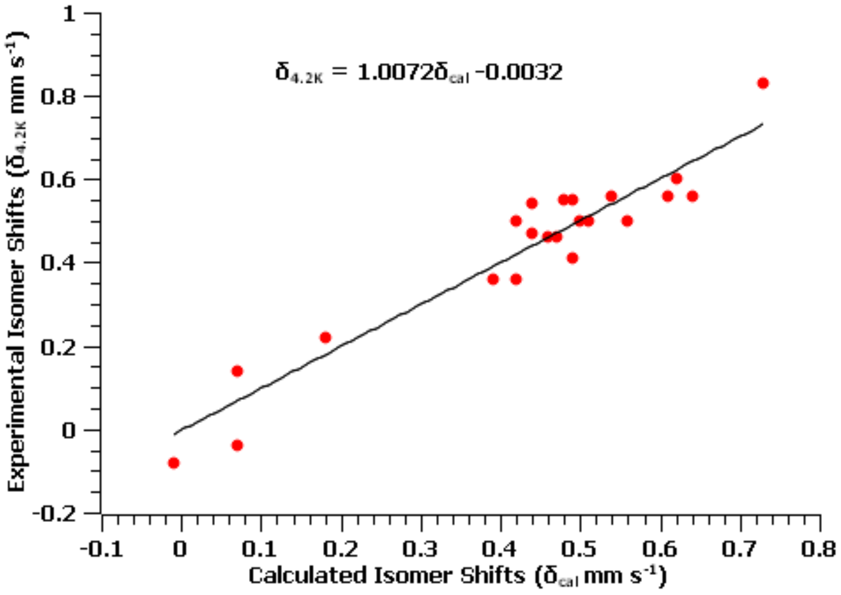

Figure S2. Correlation between the calculated and the experimental isomer shifts for the 19 sample Fe complexes (totally 30 Fe sites) given in Table S1.

This linear fitting yields the slope A = 1.007, which is almost the ideal value 1.0; and the intercept B = –0.003 mm s^{-1}, which is also around the ideal value zero. The correlation coefficient of the fitting is r = 0.947 with a standard deviation of SD = 0.067 mm s^{-1}.

2. OLYP-D3(BJ) Calculated Quadrupole Splittings for the Sample Fe complexes

The quadrupole splitting (ΔE_Q) of an Fe atom arises from the non-spherical nuclear charge distribution in the I = 3/2 excited state, and is proportional to the electric field gradient (EFG) at the Fe nucleus which can be calculated directly from ADF. The EFG tensor (V) is diagonalized and its eigenvalues are reordered so that $|V_{zz}| \geq |V_{xx}| \geq |V_{yy}|$. The asymmetry parameter η is then defined as

$$\eta = |(V_{xx} - V_{yy})/V_{zz}| \qquad \text{(S3)}$$

Finally the quadrupole splitting for ^{57}Fe of the nuclear excited state (I = 3/2) can be calculated as

$$\Delta E_Q = \tfrac{1}{2} eQV_{zz}(1 + \eta^2/3)^{1/2} \qquad \text{(S4)}$$

where e is the elementary positive charge (*i.e.* an electron has a charge of $-1e$), Q is the nuclear quadrupole moment (0.16 barns in ADF calculations) of Fe.

We obtained the calculated quadrupole splitting values ($\Delta E_{Q(cal)}$) for the 19 sample Fe complexes in ADF output, and compared them with the corresponding experimental values ($\Delta E_{Q(exp)}$) in **Table S1**. The linear correlation (see **Figure S3**) between the calculated and the observed ΔE_Q absolute values based on the equation

$$|\Delta E_{Q(exp)}| = A\,|\Delta E_{Q(cal)}| + B \qquad \text{(S5)}$$

yields A = 0.980, which is also near the ideal value 1.0; and B = –0.041 mm s^{-1}, which is also close to the ideal value zero. The correlation coefficient is r = 0.937 with the standard deviations SD = 0.301 mm s^{-1}. Therefore, the calculated quadrupole splittings correlate well with the corresponding experimental values.

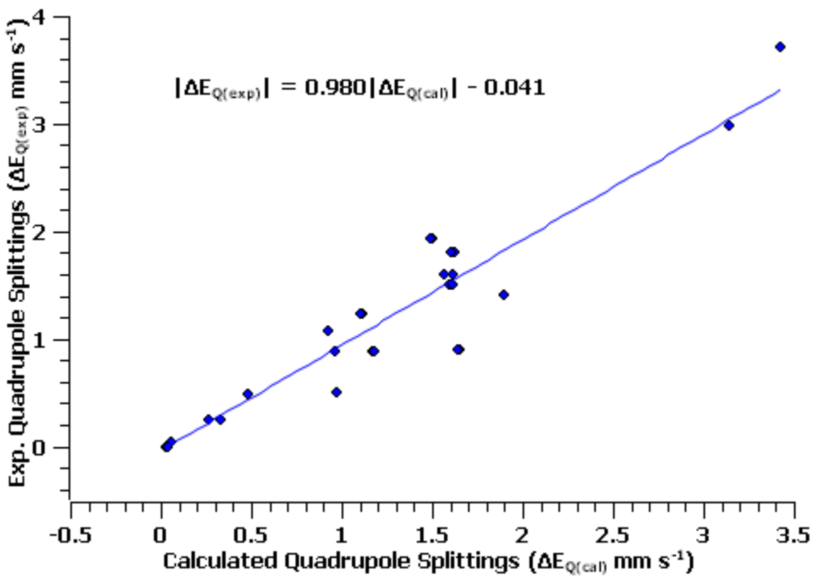

Figure S3. Correlation between the calculated and the experimental quadrupole splittings for the 19 sample Fe complexes (30 Fe sites in total) given in Table S1.

Table S1. OLYP-D3(BJ) Calculated and Experimental Isomer shifts (δ_{cal} and $\delta_{4.2K}$, mm s^{-1}) and Quadrupole Splittings ($\Delta E_{Q(cal)}$ and $\Delta E_{Q(exp)}$, mm s^{-1}) of a set of $Fe^{2.5+}$, Fe^{3+}, $Fe^{3.5+}$ and Fe^{4+} Complexes Used in the Simple Linear Regression to Get the Linear Correlation between the Calculated Electron Density on $Fe^{2.5+,3+,3.5+,4+}$ Nuclei ρ(0) and the Corresponding Experimental Isomer Shift Values at 4.2 K.

			ox-	experiment				Calculated			
complex	CSD ID [a]	S_{total}	state	T	δ_T	$\delta_{4.2K}$	$\Delta E_{Q(exp)}$	ρ(0)	δ_{cal}	$\Delta E_{Q(cal}$	$\eta_{(cal)}$
$Fe_2(salmp)_2^-$	KASGAM	9/2	+2.5	297	0.71	0.83	1.08	11819.54	0.73	0.92	1.00
			+2.5	297	0.71	0.83	1.08	11819.54	0.73	0.92	0.98
$Fe(bipy)_2Cl_2^+$	CAVDOS05	5/2	+3	RT	0.42	0.54	0.24	11820.39	0.44	0.26	0.93
FeF_6^{3-}	TUKBOQ	5/2	+3	297	0.49	0.61	0.00	11819.85	0.62	0.03	0.32
$FeCl_6^{3-}$	DALLIL	5/2	+3	78	0.53	0.56	0.04	11820.10	0.54	0.06	0.12
$FeCl_4^-$	MICYFE10	5/2	+3	RT	0.24	0.36	0.00	11820.55	0.39	0.04	0.23
$Cl_3FeOFeCl_3^{2-}$	FACTEI	0	+3	77	0.33	0.36	1.24	11820.46	0.42	1.11	0.10
			+3	77	0.33	0.36	1.24	11820.46	0.42	1.10	0.06
$Fe_2O(OAc)_2(HBpz_3)_2$	CACZIP10	0	+3	77	0.52	0.55	1.60	11820.24	0.49	1.61	0.63
			+3	77	0.52	0.55	1.60	11820.26	0.48	1.57	0.64
$Fe_2(OH)(OAc)_2(HBpz_3)_2$	COCJIN	0	+3	77	0.47	0.50	0.25	11820.21	0.50	0.26	0.62
			+3	77	0.47	0.50	0.25	11820.19	0.51	0.33	0.47
$Fe_2O(OAc)_2(Me_3TACN)$	DIBXAN10	0	+3	4.2	0.47	0.47	1.50	11820.39	0.44	1.59	0.73
			+3	4.2	0.47	0.47	1.50	11820.39	0.44	1.61	0.69
$Fe_2O(OAc)_2(bipy)_2Cl_2$	VABMUG	0	+3	120	0.37	0.41	1.80	11820.23	0.49	1.62	0.59
			+3	120	0.37	0.41	1.80	11820.24	0.49	1.60	0.51
$Fe_2(salmp)_2$	KASFOZ	0	+3	297	0.44	0.56	0.88	11819.87	0.61	1.17	0.53
			+3	297	0.44	0.56	0.88	11819.87	0.61	1.18	0.53
Fe(II)Fe(III)BPMP$(OPr)_2$	GATFOW	1/2	+3	55	0.48	0.50	0.50	11820.02	0.56	0.97	0.25
$Fe_2O_2(6TLA)_2^{2+}$	YOCKAC	0	+3	4.2	0.50	0.50	1.93	11820.46	0.42	1.50	0.74
			+3	4.2	0.50	0.50	1.93	11820.46	0.42	1.49	0.73
$Fe_2O(Me_3TACN)_2(Cl_4cat$	YOHMOX	0	+3	4.2	0.46	0.46	1.41	11820.32	0.46	1.90	0.95
			+3	4.2	0.46	0.46	1.41	11820.32	0.47	1.90	0.95
$Fe_2(Cat)_4(H_2O)_2^{2-}$	TEMKUR	0	+3	4.2	0.56	0.56	0.90	11819.79	0.64	1.65	0.63
			+3	4.2	0.56	0.56	0.90	11819.78	0.64	1.64	0.61
Fe(OEC)Cl [b]	SUMWUS	1	+3	77	0.19	0.22	2.99	11821.15	0.18	3.14	0.02
$Fe_2O_2(5\text{-}Et_3\text{-}TPA)_2^{3+}$	DEKNOW	3/2	+3.5	4.2	0.14	0.14	0.49	11821.49	0.07	0.48	0.50
			+3.5	4.2	0.14	0.14	0.49	11821.49	0.07	0.48	0.49
$Fe(OEC)C_6H_5$	SUMXED	1	+4	77	−0.11	−0.08	3.72	11821.73	-0.01	3.42	0.07
$FeCl(\eta^4\text{-MAC*})^-$	JESGUJ	2	+4	4.2	−0.04	−0.04	−0.89	11821.50	0.07	0.96	0.32

[a] The complex ID's in the Cambridge Structural Database (CSD). [b] In this complex, the Fe^{3+} site is in intermediate spin state AF-coupling with the radical on the porphyrin ring.

References in Supporting Infomation

(1) Han, W.-G.; Liu, T.; Lovell, T.; Noodleman, L. DFT Calculations of ^{57}Fe Mössbauer Isomer Shifts and Quadrupole Splittings for Iron Complexes in Polar Dielectric Media: Applications to Methane Monooxygenase and Ribonucleotide Reductase. *J. Comput. Chem.* **2006,** *27*, 1292-1306.

(2) Liu, T.; Lovell, T.; Han, W.-G.; Noodleman, L. DFT calculations of isomer shifts and quadrupole splitting parameters in synthetic iron-oxo complexes: Applications to methane monooxygenase and ribonucleotide reductase. *Inorg. Chem.* **2003,** *42*, 5244-5251.
